# Examination of How the Affordable Care Act Influenced Use of Lower-Acuity Emergency Department Services

**DOI:** 10.51894/001c.7021

**Published:** 2018-09-26

**Authors:** Joe Haddad, Kyle Fink, Katherine Pitus

**Affiliations:** 1 Emergency Medicine Faculty, Ascension Macomb-Oakland Hospital; 2 Emergency Medicine Faculty, Cox Medical Center, South Springfield, MO; 3 Emergency Medicine Program Director, Ascension Macomb-Oakland Hospital

**Keywords:** healthcare use choices, lower-acuity emergency cases, emergency department services, affordable care act

## Abstract

**CONTEXT:**

The Affordable Care Act (ACA) was implemented to make insurance accessible and reduce healthcare costs. The purpose of this study was to examine for changes in the use of lower-acuity types of Emergency Department (ED) services at two suburban Detroit facilities before, and after implementation of the ACA.

**METHODS:**

A retrospective chart review of patients presenting to the ED at a two-campus suburban hospital system was performed over two 18-month pre- and post-ACA periods. The authors completed a review of electronic health record data and used ICD-9 code and ED visit billing and collections data. Sample patients included those who had received lower-acuity ED care within the two designated time periods. A total of 16 lower-acuity ICD-9 codes were included to gauge pre and post changes in use of ED services.

**RESULTS:**

The authors identified 2,099 patients meeting study criteria during the pre-ACA period and 2,158 patients within the post-ACA period. A subgroup of 166,483 ED patients received care during the pre-ACA time period and 179,879 post-ACA. There was no statistically significant difference between the volume of lower-acuity ED visits during the two analytic periods (i.e., 1.26% seen pre-ACA implementation and 1.20% seen post-ACA). (p = 0.420) Neither did the absolute number of all ED visits significantly change. As could be anticipated, however, the proportion of self-pay patients pre-ACA significantly decreased from 506 (24.1%) to 191 (8.9%) post-ACA. (p < 0.001) Medicaid HMO payments also increased significantly from 824 visits pre-ACA to 1,086 visits post-ACA. (p < 0.001) In addition, Blue Cross coverage increased from 54 visits pre-ACA to 98 visits post-ACA. (p < 0.001)

**CONCLUSIONS:**

In summary, our results revealed no significant change in the absolute volume of all ED visits or proportions of lower-acuity ED visits between the pre- and post-ACA periods. As the authors had anticipated, pre and post changes in the number of self-pay patients and those with certain types of insurance coverage were dramatic. The authors conclude that changes in lower-acuity visits to the ED in these study settings had not decreased as envisioned by ACA developers. Future studies with larger longitudinal samples are warranted to more fully investigate the longer-term implications of the ACA on use of ED services.

## INTRODUCTION

An extensive ongoing debate in our country has ensued related to how to make health insurance coverage more readily available for our nation’s uninsured population.^1–3^ In 2010, the Affordable Care Act (ACA) was developed with several overall goals, two of which were to make insurance more accessible to those without coverage and reduce overall healthcare costs.^4^ The primary motivation for this legislation was a presumption that once the majority of people across the nation had medical insurance, they would be more likely to utilize preventive care and meet their healthcare needs in lower cost primary care settings.^2,5–7^

Similar to several state legislative changes, the ACA legislation has emphasized enhancing the availability and use of primary care services to curb disease processes before they require emergency department (ED) visits, hospital admissions and extensive treatments and procedures.^8–10^ To date, researchers have found mixed results concerning healthcare service use patterns in select settings and regions of the country. Patient behaviors have been found to vary by type of insurance coverage and the patients’ baseline health profiles, with sicker patients tending to take advantage of covered services earlier. Healthcare service use patterns have also been found to vary with the states’ current demographics, how the ACA is advertised, and whether the state expands its Medicaid coverage or focuses on expanding its health insurance marketplace.^1,2,6,7,11^

Various measures have been implemented (e.g., scholarships, loan forgiveness, financial bonuses) to incentivize primary care physicians participating in state-administered Medicaid programs.^11^ Additionally, healthcare policy makers were hopeful patients who had established relationships with a primary care provider (PCP) would decompress the nation's taxed ED and reduce healthcare costs.^2,3,9,12,13^

### Purpose of Study

The Michigan-based authors aimed to examine what pre- and post-ACA implementation changes may have occurred in the use of lower-acuity ED services in two community-based settings. The authors utilized de-identified electronic health record (EHR) data from an 18-month period before ACA implementation compared to 18 months after implementation. The EHR data were extracted from the hospital billing department Research Coordinator for the indicated periods from two Ascension Health campuses in the suburban Detroit area.

More specifically, the study questions asked included the following: (1) “To what extent has the ACA influenced relative changes in the volume of lower-acuity ED visits?” (2) “Have overall ED utilization rates changed under the ACA, as measured by absolute number of ED visits?” and (3) “What changes in payer mix (Medicaid, Medicare, uninsured, private pay, etc.) have occurred from before and after implementation of the ACA?” The authors had hypothesized that despite the best intentions of the ACA, the utilization of lower-acuity ED services would still increase despite decreases in the number of uninsured patients.^2,4,9^

There were also several longer-term goals for this study. First, the authors expected that the area emergency medicine community would be able to use this knowledge to evaluate how the ACA had affected their respective series of Michigan ED facilities. Ideally, national emergency medicine leaders and policy makers would also be able to utilize results from these types of studies to inform their decisions pertaining to future legislation and policy reform initiatives. Additionally, hospital administrations would be able to utilize these types of results to make more strategic financial adjustments for emerging reimbursement trends.

## METHODS

### Study Design

This was a retrospective EHR chart review of lower-acuity patients presenting to the ED at St. John Macomb-Oakland Hospital, including both Oakland and Macomb centers, over two 18-month periods. January 1st, 2012 - June 30, 2013 was observed as the pre-ACA implementation period, beginning two years before the implementation of the ACA on January 14, 2014. January 1st, 2015 - June 30, 2016 represented the post-ACA implementation period, beginning approximately one year after initial implementation. De-identified data concerning patients’ insurance coverage payment during both periods were also compared to analyze the reimbursement question. The authors’ institutional review board had approved the study protocol before any data collection began.

### Statistical Analyses

Univariate between-group sample subgroup comparisons were completed by the authors’ healthcare system PhD-trained research coordinator (cited in Acknowledgement section) using Chi squared test procedures with all categorical variables.^14^ The study analyst conducted all statistical analyses using SPSS version 19.0.^15^ He observed a coefficient Alpha p-value of 0.05 or less to indicate statistical significance.

### Sample Settings

St. John Macomb Hospital and St. John Oakland Hospital merged in 2007 and became St. John Macomb-Oakland Hospital. As of June 2018, the hospital campuses were renamed within the same system to Ascension Macomb-Oakland Hospital. This healthcare system is located on two campuses, with 535 beds total. St. John Ascension Oakland campus, located in Madison Heights MI, is a 159-bed acute care teaching hospital with approximately 34,000 ED visits per year. St. John Ascension Macomb campus Hospital, located in Warren MI, is a 376-bed facility with approximately 78,000 ED visits per year.

### Data collection and processing

De-identified study data were all extracted from the authors’ EHR chart review. The data for study analyses were of several types including ICD-9 diagnosis codes,^16^ ED visit billing and collections data concerning eligible patients’ insurance coverage and ED service utilization. Sample patients included all those treated at Ascension Macomb and Oakland Emergency Departments during the identified pre- and post-periods, specifically with, or without, one of the 16 lower-acuity ICD-9 discharge diagnosis codes listed in Table 1. These 16 lower-acuity ICD-9 codes were utilized to gauge changes in use of ED services between lower-acuity and all other patient sample subgroups.

**Table:** Table 1 Selected Study Lower-acuity ICD-9 Codes

Final ICD-9 Diagnosis Code	Final Diagnosis Description
460	Acute nasopharyngitis [common cold]
461	Acute sinusitis
461.8	Other acute sinusitis
461.9	Acute sinusitis, unspecified
462	Acute pharyngitis
465	Acute upper respiratory infections of multiple or unspecified sites
472	Chronic pharyngitis and nasopharyngitis
472.1	Chronic pharyngitis
473	Chronic sinusitis
473.8	Other chronic sinusitis
473.9	Unspecified sinusitis (chronic)
487.1	With other respiratory manifestations
79.99	Unspecified viral infection
487	Influenza
68.1	Medication Refill
525.9	Dental Pain

## RESULTS

As depicted in Table 2, the authors identified a total of 2,099 (1.3% of total period patients) lower-acuity patients meeting study inclusion criteria during the pre-ACA period and 2,158 (1.2%) lower-acuity patients in the post-ACA period. The total numbers of low acuity ED patients treated during the pre-ACA period was 166,483 and 179,879 low acuity patients during the post-ACA period. Unfortunately, specific patient-level test results and socio-demographic data were not available in the data sets as extracted.

**Table:** Table 2 Pre- and Post-ACA Changes in Study ICD-9 Code Claims

**ICD Code**	**Description**	**number pre-ACA**	**number post-ACA**	**Percent change Pre ACA to Post-ACA**	**p value**
460	Acute nasopharyngitis [common cold]	4	6	50.0	0.75
461	Acute sinusitis	2	0	-100.0	0.24
461.8	Other acute sinusitis	0	7	700.0	**0.02**
461.9	Acute sinusitis, unspecified	188	73	-61.2	**< 0.001**
462	Acute pharyngitis	799	355	-55.6	**< 0.001**
465	Acute upper respiratory infections of multiple or unspecified sites	0	702	7020.0	**< 0.001**
472	Chronic pharyngitis and nasopharyngitis	1	0	-100.0	0.95
472.1	Chronic pharyngitis	0	0	0.0	.0.99
473	Chronic sinusitis	0	0	0.0	.0.99
472.8	Other chronic sinusitis	1	1	0.0	1.0
473.9	Unspecified sinusitis (chronic)	240	106	-55.8	**< 0.001**
79.99	Unspecified viral infection	42	596	1319.0	**< 0.001**
487	Influenza	0	0	0.0	1.0
68.1	Medication Refill	158	96	-39.2	**< 0.001**
525.9	Dental Pain	664	216	-67.5	**< 0.001**
	**TOTAL**	**2,099**	**2,158**		

Although there was certainly a change between the specific ICD-9 and ICD-10 billing codes used during these two analytic periods (ICD-10 took effect October 1, 2015), the analyst found no statistical pre-post differences between the proportions of lower-acuity ED visits (p = 0.420). Furthermore, there was no statistical change found in ED utilization patterns as measured by absolute number of lower-acuity visits. In fact, the total number of patients seen during the post-ACA period slightly increased by 13,396. However, we did identify a statistical decrease in the low acuity visits at the Oakland campus by 0.2% (p = 0.001) as compared to no statistical change in the Macomb campus low acuity visits. (Table 3) We have since concluded that this may be due to the higher relative proportion of self-pay patients visiting the Oakland campus before the ACA. (Table 4)

**Table:** Table 3 Pre- and Post-ACA Visit Changes by Campus

	Total ED Visits Pre-ACA	Pre-ACA Low Acuity (%)	Total ED Visits Post-ACA	Post-ACA Low Acuity (%)	p value
Oakland	55293	924 (1.6%)	54579	755 (1.4%)	**< 0.001**
Macomb	111190	1175 (1.2%)	125303	1363 (1.1%)	0.47
p value		**< 0.001**		**< 0.001**	

<b>Bold</b> p values are statistically significant

**Table 4:** Pre- and Post-ACA Changes in Insurance Coverage by Campus

	Insurance Type	Pre-ACA percent total patients	Post-ACA percent total patients	P value
Oakland Hospital	Medicaid HMO	18.0%	10.1%	**0.006**
Macomb Hospital	Medicaid HMO	17.5%	29.5%	**<0.001**
Oakland Hospital	Self-Pay	15.8%	7.7%	**<0.001**
Macomb Hospital	Self-Pay	12.6%	10.8%	**0.003**
Oakland Hospital	Medicare	3.4%	2.3%	0.25
Macomb Hospital	Medicare	4.6%	3.3%	0.82
Oakland Hospital	Medicaid	2.8%	3.5%	0.62
Macomb Hospital	Medicaid	2.1%	2.4%	0.77
Oakland Hospital	Blue Care Trust	4.2%	4.6%	0.83
Macomb Hospital	Blue Care Trust	0.2%	0.9%	0.21
Oakland Hospital	Blue Cross	0.4%	0.4%	0.72
Macomb Hospital	Blue Cross	0.4%	0.7%	0.11
Oakland Hospital	Blue Care Network	2.2%	2.4%	0.54
Macomb Hospital	Blue Care Network	3.3%	6.2%	0.07
Oakland Hospital	HAP	1.5%	1.5%	0.94
Macomb Hospital	HAP	4.8%	5.4%	0.46
Oakland Hospital	Other	3.8%	4.8%	0.62
Macomb Hospital	Other	2.4%	3.4%	0.68

<b>Bold</b> p values are statistically significant

As might be expected, the overall proportion of self-pay total sample ED visit patients went down dramatically after implementation of the ACA. (Table 5) The number of self-pay patients before the ACA was 506 (24.1% of eligible sample patients) compared to 191 (8.9%) post-ACA. (p < 0.001) Proportions of certain types of insurance coverage, particularly Medicaid HMO and Blue Cross, also went up significantly post-ACA. (both p < 0.001) Medicaid HMO coverage increased from 824 visits pre-ACA to 1086 visits post-ACA, and Blue Cross from 54 visits pre to 98 visits post ACA. (p each < 0.001)

**Table:** Table 5 Pre- and Post-ACA Overall Changes in Insurance Coverage

**Insurance Coverage**	**Pre-ACA (%)** **(n = 2,099)**	**Post-ACA (%)** **(n = 2,158)**	**p value**
Self-Pay	506 (24.1%)	191 (8.9%)	**< 0.001**
Medicaid HMO	824 (39.3)	1,086 (50.3)	**< 0.001**
Medicare	154 (7.3)	135 (6.3)	0.18
Medicaid	171 (8.1)	207 (9.6)	0.08
Blue Care Trust	115 (5.5)	131 (6.1)	0.40
Blue Cross	54 (2.6)	98 (4.5)	**< 0.001**
Blue Care Network	48 (2.3)	54 (2.5)	0.64
Health Alliance Plan	32 (0.2)	33 0.2)	0.47
Other	107 (5.1)	117 (5.4)	0.63

<b>Bold</b> p values are statistically significant

## DISCUSSION

These results prompted the authors to question why the overall absolute number of ED visits, including lower-acuity visits, did not significantly decrease after we could confirm that more patients had achieved coverage under the ACA. Clearly, these results are similar to the general findings of several studies that the relationship between health insurance coverage and primary care/ED visit care continues to be very complex.^1,5,7,11^ Although our results demonstrate that the overall ED service use of Medicaid HMO and Blue Cross beneficiaries went up significantly under the ACA, many PCP continue to not accept Medicaid patients.^7^ Additionally, the co-payments expected for many patients (including those with unpaid pre-ACA balances) has continued to occur in many primary care office settings.^1,3,9^

Changes in ICD-9 to ICD-10 diagnostic codes during the total study period may have been an additional factor possibly skewing our results. During the post-ACA period, there were new lower-acuity ICD-10 codes added along with changes in which billing codes were used more frequently. (Table 2) For example, the new set of ICD-10 codes included a diagnosis code 465 “Acute upper respiratory infections of multiple or unspecified sites.” This catch-all diagnosis code was entered for 702 patients in the post-ACA period. (Figure 1 and Table 2) This code wording change appears to have influenced the numbers of some cases away from previous ICD-9 codes. Additionally, there was an increased post-ACA usage of ICD-10 code 79.99 “Unspecified viral infection.” (Table 2) Interestingly, dental pain (ICD-10 code 525.9) did see a significant drop from pre-ACA to post-ACA period, specifically, a 67.5% drop from 664 patients to 216. (Table 2) We have no well-defined reason to account for this dramatic decrease given that there is no requirement or mandate under the ACA to provide coverage for dental services. It is also unclear as to where some patients actually sought dental care during the post-ACA analytic period. There were no new openings of emergent care dental clinics in the immediate area of the two sample campuses, although increased PCP utilization was certainly possible.

**Figure:**
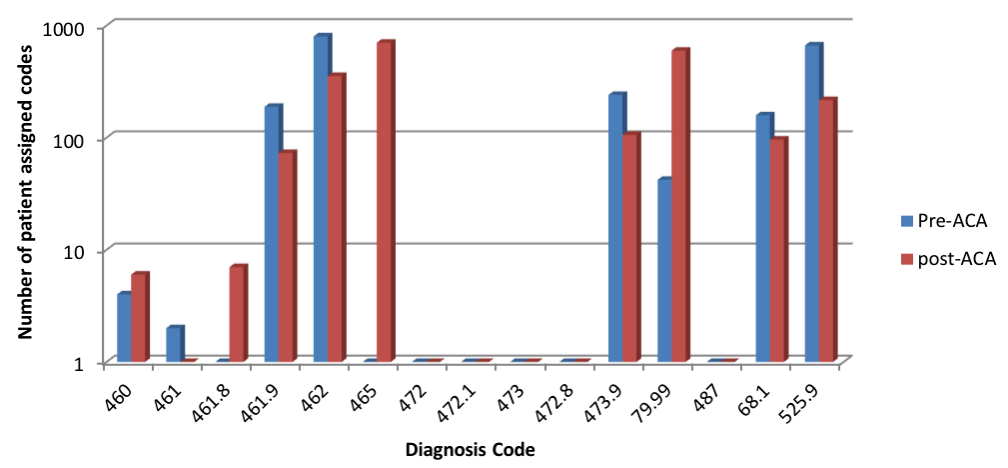
Figure 1 Pre- and Post-ACA Changes in Lower-acuity Code Claims

### Study Limitations

The data collected from this convenience sample of patients came from two Michigan suburban healthcare campuses. A larger heterogeneous sample of ED patients (e.g., also including rural and urban settings) may have improved the generalizability of our results to other ED settings.^3,7^ Furthermore, timely access to primary and specialty care services involving Medicaid has still been shown to be inconsistent, leaving many patients with few realistic healthcare alternatives to the ED.^9,11,13^ In addition, since some of the previously-uninsured patients in this sample may have been accustomed to receiving most all of their healthcare services in the ED, perhaps so much so that any such pre-ACA tendencies may take an indeterminate amount of time to change.^1,6,11,13^

Our extracted study data did indicate the type of insurance used during ED visits, but not regarding any possible deductible amounts. It can be presumed that those acquiring high-deductible health plans via the ACA marketplace would seek care for lower acuity complaints outside of the ED, making them “better” overall healthcare consumers. Since this study did not stratify those patients from those with higher deductible plans, further investigation with detailed co-payment data and patient socio-demographic data is certainty warranted.

## CONCLUSIONS

A primary goal of the ACA was to replace use of ED utilization care for lower-acuity conditions with non-urgent primary care visits.^2,3,5^ The legislation was partially based on a belief that providing increased insurance coverage would improve patients’ access to primary care, thus substituting lower-acuity ED visits with office-based visits.

Our results revealed no significance changes in the absolute number of lower-acuity ED visits between the pre- and post-ACA analytic periods. Neither was there an overall significant change noted in number of lower-acuity ED visits out of the totality of period ED visits. As might be expected, however, these study results demonstrate a dramatic proportionate decrease in self-pay ED patients as well as substantial increases in certain types of insurance coverage.

We conclude from these results that the utilization of ED services for lower-acuity conditions increased slightly in these two Michigan settings and that the idealized outcomes (i.e., greater primary care coverage for lower-acuity conditions) envisioned by ACA proponents were not readily identified. Further studies with larger longitudinal analytic samples are warranted in these and other settings to more fully gauge the longer-term influence of the ACA on consumption patterns for ED services across the nation.

### Conflict of Interest

The authors declare no conflict of interest.
